# Supplementary material for: Bound states in the continuum based on the total internal reflection of Bloch waves
Source: Natl Sci Rev. 2022 Mar 4;10(1):nwac043. doi: 10.1093/nsr/nwac043 (PMC9910412; doi:10.1093/nsr/nwac043)
Supplement: nwac043_Supplemental_Files [file nwac043_supplemental_files.zip › SI_for_TIR-based_BICs.pdf]

Supplementary Information

for

Bound States in the Continuum Based on the Total Internal Reflection of Bloch Waves

Peng Hu[1,†], Chongwu Xie[1,†], Qianju Song[1], Ang Chen[2], Hong Xiang[1,3], Dezhuan Han[1,*], and Jian Zi[2,*]

[1] College of Physics, Chongqing University, Chongqing 401331, China

[2] State Key Laboratory of Surface Physics, Key Laboratory of Micro- and Nano-Photonic Structures (Ministry of Education) and Department of Physics, Fudan University, Shanghai 200433, China

[3] Chongqing Key Laboratory for Strongly Coupled Physics, Chongqing 401331, China

[*]**Corresponding authors.** Email: dzhan@cqu.edu.cn and jzi@fudan.edu.cn
[†]Equally contributed to this work.

Dispersion relation for a 1D photonic crystal

For a binary photonic crystal (PhC), the dispersion relation relates the frequency ω, the normal component of the wave vector k_z, and the Bloch wave vector k_x. For TE waves, it is given by [1]:

$$\cos k_x a = \cos k_{1x}(a-d)\cos k_{2x}d - \tfrac{1}{2}(\eta + 1/\eta)\sin k_{1x}(a-d)\sin k_{2x}d, \qquad \text{(S1)}$$

where $\eta = k_{1x}/k_{2x}$, $k_{ix} = \sqrt{\varepsilon_i k_0^2 - k_z^2}$ $(i = 1, 2)$, $k_0 = \omega/c$, and c is the speed of light in free space. For fixed k_x and ω, there are a finite number of propagating states with $k_z^2 > 0$ and an infinite number of evanescent states with $k_z^2 < 0$ in the PhC. The isofrequency contour for the propagating states at a fixed ω can be obtained by Eq. S1, whereas the frequency ranges with different number of propagating Bloch modes (N_p) can be simply obtained by setting $k_z = 0$ in Eq. S1 and counting the number of bands below this frequency. Additionally, the frequency ranges with a different number of radiation channels (N_r) in free space can be directly obtained by folding the light line.

Method used to obtain polarization states of the *m*th-order diffraction

The electric field **E** and magnetic field **H** of guided resonance in a PhC slab can be simulated by the finite element method. For the guided resonances in the frequency range where there are multiple propagating diffraction orders ($N_r > 1$), multiple propagating plane waves radiate to different directions in free space, and thus the polarization directions of the corresponding far-field radiation cannot be directly defined. It is essential to extract these nonzero propagating-wave amplitudes from the far-field radiation. Using the Bloch theorem, the far-field component of the *m*th-order diffraction can be obtained by $\mathbf{E}_m(\mathbf{k}_{\|}) = 1/a\int_{x_0}^{x_0+a}\mathbf{E}e^{-i(k_x+mG)x}dx$ for the electric field and $\mathbf{H}_m(\mathbf{k}_{\|}) = 1/a\int_{x_0}^{x_0+a}\mathbf{H}e^{-i(k_x+mG)x}dx$ for the magnetic field, where integrations are performed on the horizontal plane away from the PhC slab. Thus, we can evaluate separately the radiation power, $P_m = 2\int_{x_0}^{x_0+a}\mathbf{S}_m\cdot\hat{z}dx$, per unit cell from the *m*th-order diffraction of the guided resonance, where the multiple of two comes from the symmetry of the structure in the *z* direction and $\mathbf{S}_m = 1/2\,\mathrm{Re}\left(\mathbf{E}_m\times\mathbf{H}_m^*\right)$ is the time-averaged Poynting vector. The quality factor that accounts for the radiative loss from the *m*th-order diffraction can then be obtained by $Q_m = \omega U_{\mathrm{eff}}/P_m$, where U_{eff} is the stored energy in the PhC slab and can be directly calculated by numerical simulations.

To characterize the polarization states of the *m*th-order diffraction, we decompose $\mathbf{E}_m(\mathbf{k}_{\|})$ into two orthogonal components on the *s*–*p* plane: $\mathbf{E}_m\left(\mathbf{k}_{\|}\right) = t_m^s\left(\mathbf{k}_{\|}\right)\hat{e}_m^s + t_m^p\left(\mathbf{k}_{\|}\right)\hat{e}_m^p$, where $\hat{e}_m^s = \hat{z}\times\mathbf{k}_m/\left|\hat{z}\times\mathbf{k}_m\right|$, $\hat{e}_m^p = \mathbf{k}_m\times\hat{e}_m^s/\left|\mathbf{k}_m\times\hat{e}_m^s\right|$, and $\mathbf{k}_m = (k_x+mG)\hat{x}+k_y\hat{y}+k_{z,m}\hat{z}$ is the wave vector of the *m*th-order diffracted wave in free space. Further, the Stokes parameters of $\mathbf{E}_m$ are employed to describe the corresponding polarization states, namely, $S_{0,m} = \left|t_m^p\left(\mathbf{k}_{\|}\right)\right|^2 + \left|t_m^s\left(\mathbf{k}_{\|}\right)\right|^2$, $S_{1,m} = \left|t_m^p\left(\mathbf{k}_{\|}\right)\right|^2 - \left|t_m^s\left(\mathbf{k}_{\|}\right)\right|^2$, $S_{2,m} = 2\,\mathrm{Re}\left[t_m^{p*}\left(\mathbf{k}_{\|}\right)t_m^s\left(\mathbf{k}_{\|}\right)\right]$ and $S_{3,m} = 2\,\mathrm{Im}\left[t_m^{p*}\left(\mathbf{k}_{\|}\right)t_m^s\left(\mathbf{k}_{\|}\right)\right]$.

Some examples of the BIC solver

Based on the generalized conditions for waveguide modes that are explained in the main text, a BIC solver for 1D PhC slab is developed. Here, we first show an example of the BIC solver in the frequency range with $N_p = 2$ and $N_r = 1$, which is indicated by the gray

shaded region in **Supplementary Fig. 1a**. Assuming that the total internal reflection (TIR) of TE Bloch waves is satisfied at the upper interface of the PhC slab and N modes are considered in total, by eliminating of the coefficients t_m, Eq. 6 in the main text can be rewritten as follows:

$$\sum_{n=1}^{N}(a_n e^{ik_z^{(n)}h/2}\alpha_{m,n} + r_n e^{-ik_z^{(n)}h/2}\beta_{m,n}) = 0, \quad \text{(S2)}$$

where $\alpha_{m,n} = (1-\rho_{m,n})\tilde{u}_m^{(n)}$, $\beta_{m,n} = (1+\rho_{m,n})\tilde{u}_m^{(n)}$, $\rho_{m,n} = k_z^{(n)} / k_{z,m}$ .and $\tilde{u}_m^{(n)} = \overleftrightarrow{\mathbf{X}}_{mn}$. The TIR condition in Eq. 9 in the main text can be expressed as follows:

$$\sum_{n=1}^{N}(a_n e^{ik_z^{(n)}h/2} \pm r_n e^{-ik_z^{(n)}h/2})\tilde{u}_0^{(n)} = 0\,. \quad \text{(S3)}$$

Here, the origin of the z axis is set at the center of the PhC slab for convenience and one more equation in Eq. S2 is required if one more evanescent mode is taken into account.

Given an initial h, the reflection phase shift $\varphi_{\mathrm{r}}^{(n)} = \arg(r_n / a_n e^{-ik_z^{(n)}h})$ at the upper interface for every (k_x, ω) point can be obtained by solving Eqs. S2 and S3 with $r_n = a_n\ (n > 3)$ for all evanescent modes. In the case of $N_{\mathrm{p}} - N_{\mathrm{r}} = 1$, the phase shifts $\varphi_r^{(n)}$ are fixed at any (k_x, ω) point for different thickness h. The phase shift of the first Bloch wave $\varphi_{\mathrm{r}}^{(1)}$ is taken as example and shown in **Supplementary Fig. 1b** for a fixed k_x and a different number of evanescent Bloch waves. It is found that the convergence is very fast as the number of evanescent Bloch waves $N - N_{\mathrm{p}} \geq 2$. In other words, the positions of BICs in the k_x–ω space converge very quickly if only a few evanescent waves are considered in addition to the propagating Bloch waves. For simplicity, we choose $N - N_{\mathrm{p}} = 2$ in the solver by default to find BICs. In this way, a database of $\varphi_r^{(n)}$ for a PhC in the whole k_x–ω space can be built. Finally, for any thickness h, the total phase of a round trip for the nth propagating Bloch wave inside the PhC slab is simply $k_z^{(n)}h + \varphi_{\mathrm{r}}^{(n)}$. What the solver should do is to determine whether this phase is integer multiples of π. Moreover, when the electric field **E** and magnetic field **H** as well as ε and μ are simultaneously exchanged, Maxwell's equations will remain unchanged [2], and thus the solver can also be directly applied to TM Bloch waves. When other structural parameters of a PhC slab are fixed, the obtained TE and TM BICs for different thickness h (solid lines) are shown in **Supplementary Fig. 1c and 1d**, respectively, agreeing well with the simulated results

(dots). We further show the calculation process for the TE case in **Supplementary Video 1**, fully demonstrating the low computational complexity of this solver.

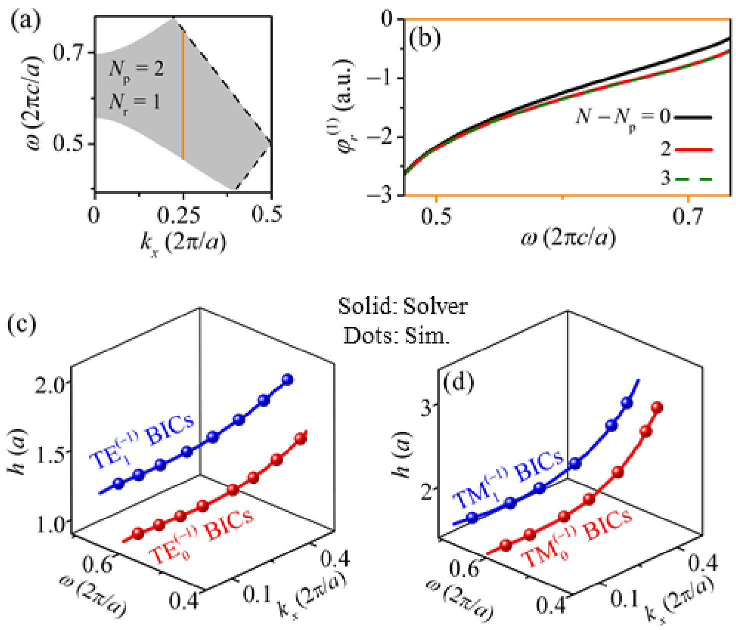

Supplementary Fig. 1 Examples of the BIC solver in the frequency range with $N_\mathrm{p} = 2$ and $N_\mathrm{r} = 1$. (a) The gray shaded region indicates the frequency range, and the black dashed lines represent the folded light line in free space. (b) Convergence of the phase shift of the first Bloch wave $\varphi_\mathrm{r}^{(1)}$ for different numbers of evanescent Bloch waves $N - N_\mathrm{p}$. This example is calculated at $k_x = 0.5\pi/a$, indicated by the orange line in (a). (c) and (d) TE and TM BICs for different thickness h. Solid lines denote the results of the BIC solver, whereas the simulated results are denoted by dots. Here, the other structural parameters are chosen as $\varepsilon_1 = 1$, $\varepsilon_2 = 4.9$, and $d = 0.5a$.

The generalized conditions for waveguide modes in PhC slabs can be applied to not only the k_x axis but also the whole Brillouin zone. Therefore, this BIC solver can work in the whole $\mathbf{k}_{||}$–ω space, where $\mathbf{k}_{||} = (k_x, k_y)$. For a 1D PhC, when k_y is not equal to zero, the bulk mode is no longer a pure TE or TM Bloch wave. Thus, for the guided resonances on the TE band in the k_x axis consisting of two TE propagating Bloch waves, the guided resonances on the corresponding TE-like band are composed of four propagating Bloch waves, i.e., two TE and two TM propagating Bloch waves, and $N_\mathrm{p} = 4$. Moreover, these guided resonance for $k_y \neq 0$ radiate to far field by both the s- and p-polarization channels, namely, $N_\mathrm{r} = 2$. In this case ($N_\mathrm{p} - N_\mathrm{r} = 2$), to find a convergent solution, an additional

condition, $r_n = a_n$ for one of the four propagating Bloch modes, is also adopted in addition to $r_n = a_n$ for all evanescent Bloch modes. In this solver, we choose $r_1 = a_1$ for the first propagating Bloch mode by default. However, this additional condition makes the phase shift $\varphi_{\mathrm{r}}^{(n)}$ at a certain ($\mathbf{k}_{\|}$, ω) point no longer a constant for different h when the TIR condition of Bloch waves is satisfied at the upper interface. Therefore, the solver has to recalculate the phase shift $\varphi_{\mathrm{r}}^{(n)}$ for different h to find BICs. Generally, BICs exist on the high symmetry lines, and an example of determination of BICs in the k_y axis in the parameter space is shown in **Supplementary Fig. 2**, which is also in full agreement with the simulated results.

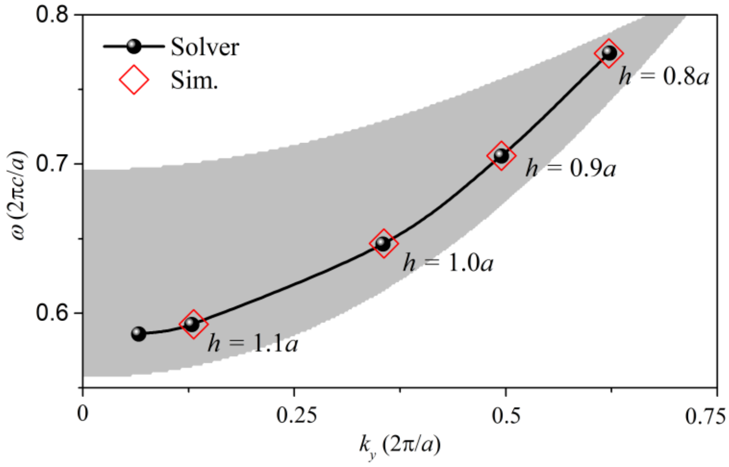

Supplementary Fig. 2 Examples of the BIC solver in the k_y axis. The gray shaded region indicates the region with $N_{\mathrm{p}} = 4$ and $N_{\mathrm{r}} = 2$. Solid line and black dots denote the results of the BIC solver, and red rhombuses indicated the simulated results for different h.

Furthermore, the generalized conditions for waveguide modes can be directly applied to the case with multiple radiation channels and thus the BIC solver can also work well to search multi-channel BICs. For the case of two radiation channels, the BIC solver can readily find multi-channel BICs in a wide range of thickness h with fast convergence, as shown in **Supplementary Fig. 3**. Similar to that shown in **Supplementary Fig. 1b**, the results converge very quickly when a few evanescent waves are considered. Examples of multi-channel BICs with h_{BIC} less than $3a$ are shown in **Supplementary Fig. 3b**, and the corresponding parameters (h_{BIC}, $k_{x,\mathrm{BIC}}$, ω_{BIC}) are also exhibited in **Supplementary Table 1** for clarity. The multi-channel BICs labelled by ① and ⑤ correspond to those in Fig. 3(a) and 3(b) in the main text, respectively.

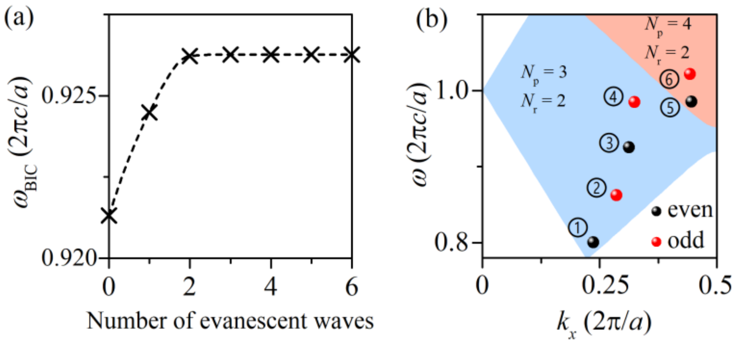

Supplementary Fig. 3. BICs with two radiation channels. (a) Convergence of ω_{BIC} for different number of evanescent modes. (b) Examples of multi-channel BICs with $h_{BIC} < 3a$. The other system parameters are chosen as $\varepsilon_1 = 1$, $\varepsilon_2 = 4.9$, and $d = 0.5a$.

Supplementary Table 1. Examples of multi-channel BICs for $h_{BIC} < 3a$.

No.	h_{BIC} (a)	$k_{x,BIC}$ ($2\pi/a$)	ω_{BIC} ($2\pi c/a$)
①	1.948	0.237	0.800
②	2.069	0.286	0.862
③	2.168	0.312	0.926
④	2.261	0.325	0.985
⑤	1.968	0.446	0.985
⑥	2.147	0.443	1.022

When there exist more radiation channels in free space ($N_r \geqslant 3$), the construction of a BIC requires that all Q factors accounting for the radiative loss towards different radiation channels, i.e., Q_0, $Q_{\pm 1}$, …, should diverge at the same point in momentum space simultaneously. This is very difficult to achieve since one needs many degrees of freedom to cancel out all these losses in different channels and search in a very large parameter space. In this sense, an algorithm with very fast convergence speed is especially useful. As examples, by only varying the thickness h, the Q factors of quasi-BICs embedded in multiple radiation channels can be maximized very quickly. A quasi-BIC for $h = 13.018a$ with 3 radiation channels is shown in **Supplementary Fig. 4.** This multi-channel quasi-BIC appear on the band of the PhC slab, as highlighted by red dots in **Supplementary Fig. 4a**. The corresponding Q factors of this band are plotted in **Supplementary Fig. 4b**, where the inset shows a zoomed-in figure near the quasi-BIC. Similar to Figs. 4 and 5 in the main text, the corresponding polarization maps (left panel) and Q factors (right panel) for the 0th- (upper), –1st- (middle), and 1st- (lower) order diffractions in the vicinity of

quasi-BIC are shown in **Supplementary Fig. 4c**. Clearly, only Q_1 diverges at a certain k_x, corresponding to one topological charge (black dot) in the polarization map for the 1st-order diffraction. The peaks of Q_0, Q_{-1} and Q_1 still deviate from each other slightly in the k_x axis since here we only optimize the slab thickness. More degrees of freedom are needed to be considered to achieve an ideal BIC. For comparison, the Q factor for the same band with h = 13.1a is also shown in **Supplementary Fig. 4b**, and there are no quasi-BICs with ultra-high Q factor.

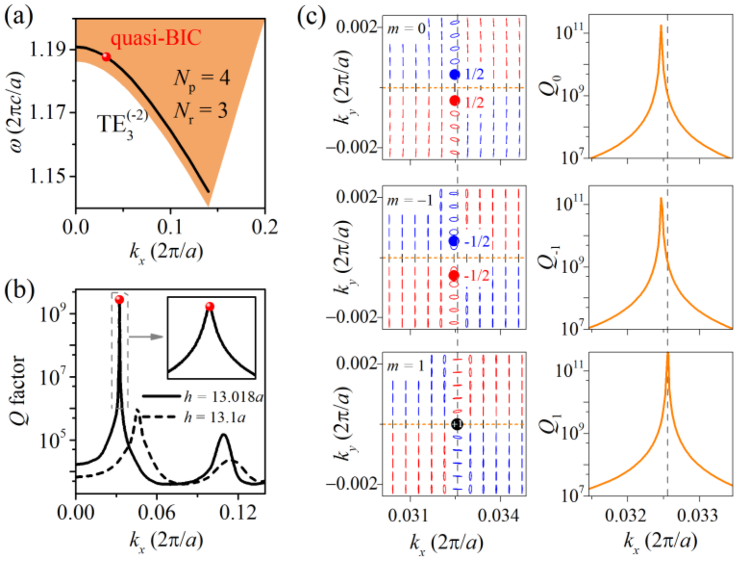

Supplementary Fig. 4. Quasi-BICs with three radiation channels. (a) Simulated band structure with $h = 13.018a$. A multi-channel quasi-BIC (red dot) exists on the band. The orange shaded region indicates the region in which there are four propagating Bloch modes ($N_\mathrm{p} = 4$) in the photonic crystal and three radiation channels ($N_\mathrm{r} = 3$) in free space. (b) Simulated Q factors of the guided resonances for $h = 13.018a$ (solid line) and $h = 13.1a$ (dashed line) in the $\mathrm{TE}_3^{(-2)}$ band. (c) Polarization maps (left panel) and Q factors (right panel) for 0th- (upper), −1st- (middle) and 1st- (lower) order diffractions in the vicinity of the quasi-BIC at $h = 13.018a$. Black (blue and red) dots indexed by the topological charge ±1 (±1/2) represent the vortex centers (circularly polarized states with LCP or RCP). The half charges lie very close to but still away from the k_x axis when the Q factor is maximized by varying h. The other system parameters are chosen as $\varepsilon_1 = 1$, $\varepsilon_2 = 4.9$, and $d = 0.5a$.

Total internal reflection of two Bloch waves

For the PhC with a small periodic index modulation, we assume that only propagating Bloch waves are considered and all evanescent Bloch waves are excluded here. We

consider the case when there are two propagating Bloch waves ($N_\mathrm{p} = 2$) and only one radiation channel in free space ($N_\mathrm{r} = 1$). For the nth TE Bloch wave in the PhC, the periodic-in-cell part of the electric field can be expanded as $u^{(n)}(x) = 1 + \tilde{u}_{-1}^{(n)} e^{-iGx} + \cdots$, where the 0th Fourier coefficient is specially set to be one. When the two TE Bloch waves impinge on the PhC interface, for $m = 0$, the boundary conditions in Eqs. 4 and 5 in the main text can be rewritten as follows:

$$\sum_{n=1}^{2} (a_n + r_n) = t_0 \tag{S4}$$

and

$$\sum_{n=1}^{2} (a_n - r_n) k_z^{(n)} = k_{z,0} t_0 \,. \tag{S5}$$

For $m = -1$, by eliminating coefficients t_m of the diffracted evanescent waves in free space, we have the following:

$$\sum_{n=1}^{2} \left(a_n \left(k_{z,-1} - k_z^{(n)} \right) + r_n \left(k_{z,-1} + k_z^{(n)} \right) \right) \tilde{u}_{-1}^{(n)} = 0 \,. \tag{S6}$$

When the total internal reflection (TIR) of two TE Bloch waves occurs, namely, $t_0 = 0$ and by combining Eqs. S4–S6, the relative incidence coefficient can be obtained and written as follows:

$$\frac{a_2}{a_1} = -\frac{1 + Z_2}{1 + Z_1} \,, \tag{S7}$$

where $Z_n = k_{z,-1} / k_z^{(n)}$ for nth propagating TE Bloch waves. The expression for the reflection coefficient is written as follows:

$$\frac{r_n}{a_n} = \frac{1 - Z_n}{1 + Z_n} \,. \tag{S8}$$

These are Eqs. 11 and 12 in the main text.

For the TM Bloch waves, the eigen wave function changes from electric to magnetic field, which can be expressed as $H_y^{(n)}(x) = v^{(n)}(x) e^{i\left(k_x x + k_z^{(n)} z\right)}$ for the nth state. Similar to the TE Bloch waves, the periodic part can be expanded as $v^{(n)}(x) = 1 + \tilde{v}_{-1}^{(n)} e^{-iGx} + \tilde{v}_1^{(n)} e^{iGx} + \cdots$. In this case, the periodic index modulation $\varepsilon(x)$ plays an important role in the boundary condition, and we adopt the first-order approximation

for the Fourier transform of $\varepsilon^{-1}(x)$, that is, $\varepsilon^{-1}(x) \approx \kappa_0 + \kappa_1 e^{iGx} + \kappa_{-1} e^{-iGx}$. The wave equation for $H_y^{(n)}(x)$ is given by:

$$\frac{\partial \varepsilon^{-1}(x)}{\partial x}\frac{\partial H_y^{(n)}}{\partial x} + \varepsilon^{-1}(x)\left(\frac{\partial^2}{\partial x^2} + \frac{\partial^2}{\partial z^2}\right)H_y^{(n)} = -k_0^2 H_y^{(n)} . \tag{S9}$$

Substituting the expansion of $H_y^{(n)}$ and $\varepsilon^{-1}(x)$ into this wave equation, we obtain

$$\kappa_1\left\{(k_x + mG)(k_x + (m-1)G) + \left(k_z^{(n)}\right)^2\right\}\tilde{v}_{m-1}^{(n)} + \kappa_{-1}\left\{(k_x + mG)(k_x + (m+1)G) + \left(k_z^{(n)}\right)^2\right\}\tilde{v}_{m+1}^{(n)}$$
$$\approx \left\{k_0^2 - \kappa_0\left[(k_x + mG)^2 + \left(k_z^{(n)}\right)^2\right]\right\}\tilde{v}_m^{(n)} . \tag{S10}$$

For $m = 0$,

$$\tilde{v}_0^{(n)} \approx \frac{\kappa_1\left[k_x(k_x - G) + \left(k_z^{(n)}\right)^2\right]\tilde{v}_{-1}^{(n)} + \kappa_{-1}\left[k_x(k_x + G) + \left(k_z^{(n)}\right)^2\right]\tilde{v}_1^{(n)}}{k_0^2 - \kappa_0\left[k_x^2 + \left(k_z^{(n)}\right)^2\right]} . \tag{S11}$$

For $m = -1$,

$$\tilde{v}_{-1}^{(n)} \approx \frac{\kappa_1\left[(k_x - G)(k_x - 2G) + \left(k_z^{(n)}\right)^2\right]\tilde{v}_{-2}^{(n)} + \kappa_{-1}\left[k_x(k_x - G) + \left(k_z^{(n)}\right)^2\right]\tilde{v}_0^{(n)}}{k_0^2 - \kappa_0\left[(k_x - G)^2 + \left(k_z^{(n)}\right)^2\right]} . \tag{S12}$$

For a vanishing index modulation ($\Delta = (\varepsilon_2 - \varepsilon_1)/\varepsilon_1 \to 0$), $k_z^{(1)}$ and $k_z^{(2)}$ approach $\sqrt{\varepsilon_{\text{eff}} k_0^2 - (k_x - G)^2}$ and $\sqrt{\varepsilon_{\text{eff}} k_0^2 - k_x^2}$, respectively, where $\varepsilon_{\text{eff}} \approx 1/\kappa_0$ is the effective permittivity for the TM case. Therefore, if k_x is not near the Brillouin zone center, $\tilde{v}_{-1}^{(1)}$ is dominant in the expansion of $H_y^{(1)}$, whereas the dominant term of $H_y^{(2)}$ is $\tilde{v}_0^{(2)}$. In this case, we only keep $\tilde{v}_0^{(n)}$ and $\tilde{v}_{-1}^{(n)}$ in the expansion of $H_y^{(n)}$ and neglect all other terms. We can then obtain $\tilde{v}_{-1}^{(n)}$ from Eqs. S11 and S12, that is:

$$\tilde{v}_{-1}^{(1)} \approx \frac{k_0^2 - \kappa_0\left[k_x^2 + \left(k_z^{(1)}\right)^2\right]}{\kappa_1\left[k_x(k_x - G) + \left(k_z^{(1)}\right)^2\right]} \quad \text{and} \quad \tilde{v}_{-1}^{(2)} = \frac{k_0^2 - \kappa_0\left[k_x^2 + \left(k_z^{(2)}\right)^2\right]}{\kappa_1\left[k_x(k_x - G) + \left(k_z^{(2)}\right)^2\right]} \approx 0 . \tag{S13}$$

When the TIR of two TM Bloch waves occurs at the PhC interface, the boundary conditions for $m = 0$ are as follows:

$$\sum_{n=1}^{2}(a_n + r_n) = 0 \tag{S14}$$

and

$$\sum_{n=1}^{2} k_z^{(n)} \left(a_n - r_n\right)\left(\kappa_0 + \kappa_1 \tilde{v}_{-1}^{(n)}\right) = 0 . \tag{S15}$$

Similar to Eq. S6, for $m = -1$, we have the following:

$$\sum_{n=1}^{2} a_n \left(k_{z,-1} \tilde{v}_{-1}^{(n)} / \varepsilon_{\mathrm{b}} - k_z^{(n)} \left(\kappa_{-1} + \kappa_0 \tilde{v}_{-1}^{(n)}\right)\right) + r_n \left(k_{z,-1} \tilde{v}_{-1}^{(n)} / \varepsilon_{\mathrm{b}} + k_z^{(n)} \left(\kappa_{-1} + \kappa_0 \tilde{v}_{-1}^{(n)}\right)\right) = 0 . \tag{S16}$$

Combining Eqs. S14–S16, the relative incidence coefficient and reflection coefficients for the TIR of two TM Bloch waves are derived and, respectively, expressed as follows:

$$\frac{a_2}{a_1} = -\frac{k_z^{(1)} \left[\varepsilon_{\mathrm{b}} k_z^{(2)} \left(\kappa_0^2 - \kappa_{-1}\kappa_1\right) + k_{z,-1} \left(\kappa_1 \tilde{v}_{-1}^{(1)} + \kappa_0\right)\right]}{k_z^{(2)} \left[\varepsilon_{\mathrm{b}} k_z^{(1)} \left(\kappa_0^2 - \kappa_{-1}\kappa_1\right) + k_{z,-1} \left(\kappa_1 \tilde{v}_{-1}^{(2)} + \kappa_0\right)\right]}, \tag{S17}$$

$$\frac{r_1}{a_1} = \frac{\varepsilon_{\mathrm{b}} k_z^{(1)} \left(\kappa_0^2 - \kappa_{-1}\kappa_1\right) - k_{z,-1} \left(\kappa_1 \tilde{v}_{-1}^{(2)} + \kappa_0\right)}{\varepsilon_{\mathrm{b}} k_z^{(1)} \left(\kappa_0^2 - \kappa_{-1}\kappa_1\right) + k_{z,-1} \left(\kappa_1 \tilde{v}_{-1}^{(2)} + \kappa_0\right)}, \tag{S18}$$

and

$$\frac{r_2}{a_2} = \frac{\varepsilon_{\mathrm{b}} k_z^{(2)} \left(\kappa_0^2 - \kappa_{-1}\kappa_1\right) - k_{z,-1} \left(\kappa_1 \tilde{v}_{-1}^{(1)} + \kappa_0\right)}{\varepsilon_{\mathrm{b}} k_z^{(2)} \left(\kappa_0^2 - \kappa_{-1}\kappa_1\right) + k_{z,-1} \left(\kappa_1 \tilde{v}_{-1}^{(1)} + \kappa_0\right)}. \tag{S19}$$

Substituting Eq. S13 into Eqs. S17–S19 and neglecting the term $\kappa_{-1}\kappa_1$, they can be simplified as $\frac{a_2}{a_1} = -\frac{1+Z_2}{1+Z_1}$ and $\frac{r_n}{a_n} = \frac{1-Z_n}{1+Z_n}$, where $Z_1 = \frac{k_{z,-1}/\varepsilon_{\mathrm{b}}}{k_z^{(1)}/\varepsilon_{\mathrm{H}}}$ and $Z_2 = C\frac{k_{z,-1}/\varepsilon_{\mathrm{b}}}{k_z^{(2)}/\varepsilon_{\mathrm{H}}}$ with $\varepsilon_{\mathrm{H}} = \kappa_0^{-1}$ and $C = \left(\left(k_z^{(2)}\right)^2 + k_x^2 - k_x G\right) \Big/ \left(\left(k_z^{(2)}\right)^2 + k_x^2 + k_x G - G^2\right)$, corresponding to Eq. 13 in the main text. Here, we use the relation $\left(k_z^{(2)}\right)^2 \approx \left(k_z^{(1)}\right)^2 - G^2 + 2k_x G$ for small index contrast in the expression of C.

Reference

1. Yariv A, Yeh P. *Optical Waves in Crystals*. New York: Wiley; 1984.
2. Born M, Wolf E. *Principles of oprics*. Cambridge: Cambridge University Press; 1999.
